# Epigallocatechin Gallate Slows Retinal Degeneration, Reduces Oxidative Damage, and Modifies Circadian Rhythms in P23H Rats

**DOI:** 10.3390/antiox9080718

**Published:** 2020-08-08

**Authors:** Lorena Perdices, Lorena Fuentes-Broto, Francisco Segura, Nicolás Cuenca, Elvira Orduna-Hospital, Isabel Pinilla

**Affiliations:** 1 Aragon Institute for Health Research (IIS Aragón), 50009 Zaragoza, Spain; lperdices@gmail.com (L.P.); ipinilla@unizar.es (I.P.); 2 Department of Pharmacology, Physiology and Legal and Forensic Medicine, Universidad de Zaragoza, 50009 Zaragoza, Spain; 3 Department of Applied Physics, Universidad de Zaragoza, 50009 Zaragoza, Spain; psegura@unizar.es; 4 Department of Physiology, Genetics and Microbiology, University of Alicante, 03690 San Vicente del Raspeig, Alicante, Spain; cuenca@ua.es; 5 Department of Ophthalmology, Miguel Servet University Hospital, 50009 Zaragoza, Spain; elvisabi14@hotmail.com; 6 Department of Ophthalmology, Lozano Blesa University Hospital, 50009 Zaragoza, Spain

**Keywords:** retinal degeneration, retinitis pigmentosa, visual function, oxidative damage, circadian rhythm, antioxidant therapy, epigallocatechin gallate, P23H rat, neurodegenerative model, green tea

## Abstract

Retinitis pigmentosa (RP) includes a group of genetic disorders that involve the loss of visual function due to mutations mainly in photoreceptors but also in other retinal cells. Apoptosis, retinal disorganization, and inflammation are common in the progression of the disease. Epigallocatechin gallate (EGCG) has been proved as beneficial in different eye diseases. Pigmented heterozygous P23H rat was used as an animal model of RP. Visual function was assessed by optomotor and electroretinogram (ERG) and circadian rhythms were evaluated by telemetry. Hepatic oxidative damage and antioxidant defenses were assessed using biochemical tests. The visual function of the EGCG P23H group was preserved, with a deterioration in the activity period and lower values in the interdaily stability parameter. Control rats treated with EGCG were less active than the sham group. EGCG increased antioxidant levels in P23H rats but reduced total hepatic antioxidant capacity by almost 42% in control rats compared to the sham group. We conclude that treatment with EGCG improves visual function and antioxidant status in P23H rats but diminishes antioxidant defenses in wild-type control animals, and slightly worsens activity circadian rhythms. Further studies are necessary to clarify the beneficial effects in disease conditions and in healthy organisms.

## 1. Introduction

The human retina is a complex structure composed of different types of cells; perfect sight requires numerous connections between neurons and glial cells. Retinal degeneration, as a result of inherited diseases, physical damage, or other pathologies, produces changes in retinal circuitry and remodeling with the loss of visual function. Inherited retinal degenerative diseases, such as retinitis pigmentosa (RP), are caused by mutations in genes involved in visual function, generating a progressive loss of photoreceptors. Despite the genotypic heterogeneity in this disease, most patients are characterized by nyctalopia, a progressive loss of peripheral visual field, and bone spicules in the fundus. With the progression of RP, changes in color vision, visual field, visual acuity (VA), and electroretinogram (ERG) are detected [1]. More than 60 genes can be affected by mutations, resulting in RP; one of the most studied genes is *RHO*, which encodes the rhodopsin protein, with more than 150 associated mutations currently identified [2]. P23H mutation is an autosomal-dominant RP commonly found in the USA [3]. In patients with this genetic mutation, the rhodopsin is retained in the endoplasmic reticulum, causing apoptosis due to the activation of the unfolded protein response and triggering an excessive production of reactive oxygen species, which contribute to the death of other retinal cells. Different transgenic animal models, including rodents and pigs, have the same mutation and similar retinal changes to RP patients [4,5].

P23H rat is one of the most used animal models. There are three known lines, depending on the speed of degeneration of retinal cells. These animals are characterized by primary rod photoreceptor degeneration, as in human RP, which continues until the complete loss and disorganization of retinal tissue. The slower progression of the disease in these animals allows the administration of long-term treatments compared to other retinal animal models [5,6]. In addition, this mutation triggers molecular and morphological changes in the inner retina, which is involved in circadian mechanisms [7]. Dysfunction in circadian rhythms have been observed in patients with RP [8].

To deeply study visual responses, in addition to measuring visual acuity, contrast sensitivity can be measured, which can be diminished even with normal visual acuity in some pathologies, such as retinal degeneration. ERG recordings provide precise information about the electrical potentials that are produced in the retina after a light stimulus, providing information about the functionality of the outer layers of the retina (photoreceptors and bipolar cells).

There is no curative treatment for RP, but new therapies could slow retinal degeneration, acting against different mechanisms that have been described in the etiology of retinal degeneration such as oxidative stress, activation of apoptotic pathways, inflammation, and glial activation or retinal vascular changes with homeostasis modification. Previous studies analyzed the levels of oxidative stress in RP in the retina [9,10,11,12,13,14,15,16,17,18,19,20,21]. Our group previously reported that the liver is under increased oxidative stress in RP [22]. As the liver has a sophisticated antioxidant system for maintaining redox homeostasis, it would be interesting to know if any antioxidant treatment against RP is powerful enough to help the liver regain redox homeostasis. Therefore, studding biomarkers of lipid peroxidation, oxidative damage to proteins, nitrosative damage, ratio of reduced glutathione to oxidized glutathione (GSH/GSSG ratio), total antioxidant capacity, and antioxidant enzymes should help with understanding the effect of the treatment in regaining redox homeostasis. Notably, the retina has non-visual functions such as the synchronization of biological rhythms. The degeneration of photoreceptors and inner retinal neurons, characteristic of RP, was previously reported to have age-related degenerative effects on the melanopsin system and is associated with weaker circadian patterns [23]. Therefore, studying biological rhythms, for example, rhythms of temperature and activity, could help with determining the effectiveness of treatments in non-visual retinal functionality.

Polyphenols, a group of substances with antioxidant effects, can be found in the diet, mainly in fruits and vegetables. The catechins are a group of polyphenolic compounds present in different teas, widely used in traditional Chinese medicine (TCM), that have demonstrated beneficial properties [24]. Epigallocatechin gallate (EGCG) is the most predominantly catechin found in green tea and has the strongest antioxidant effect; this catechin was proven to be an efficient scavenger of free radicals (including reactive oxygen and nitrogen species), a chelating agent (inhibiting some metal-dependent free radical synthesis), and a modulator of gene expression of pro-oxidant and antioxidant enzymes by transcription factors such as nuclear factor-kappa B (NF-κB) [25]. Due to its low molecular weight and water-soluble nature, EGCG is able to reach different compartments and eye tissues [26], such as the cornea, lens, and retina, to exert its antioxidant action. Previous studies demonstrated the beneficial effect of EGCG due to its antioxidant properties in ocular pathologies such as cataracts [27], dry eye [28], glaucoma [29], age macular degeneration [30], diabetic retinopathy [31], and retinal ischemia [32,33]. Polyphenols and EGCG show protective effects in retinal degeneration animal models such as N-methyl-N-nitrosourea injection [34], ischemia-reperfusion [35], and light-induced damage [36,37,38].

The aim of this study was to examine the protective effect of EGCG in the photoreceptor loss of the pigmented P23H rat. This RP model was demonstrated to be helpful for mimicking human disease and it is the most frequently used due to its progression rate [39].

## 2. Materials and Methods

### 2.1. Animals

Line 1 P23H transgenic rats (SD-Tg(Rho*P23H)1Lav) obtained from Dr. Matthew LaVail (University of California, San Francisco, CA, USA) were bred in a colony in the animal facilities of Aragon Health Sciences Institute (IACS). They were crossed with pigmented Long Evans (LE) rats (Charles River Laboratories, Barcelona, Spain) to produce pigmented heterozygous P23H × LE rats. The wild-type control group was formed by crossing Sprague–Dawley (SD) rats (Harland Ibérica, Barcelona, Spain) with LE rats.

All animals were maintained under controlled humidity (60%), temperature (23 ± 1 °C), and photoperiod (light:dark 12:12) conditions. Dry food and water were available ad libitum. All animals were housed and handled with the authorization and supervision of the Ethic Committee for Clinical Research of Aragon from the University of Zaragoza under project license PI12/14. All procedures were carried out according to the Spanish Policy for Animal Protection RD53/2013, which meets the guidelines on the ethical use of animals from the European community council (Directive 2010/63/EU), the Association for Research in Vision and Ophthalmology (ARVO) Statement for the Use of Animals in Ophthalmic and Vision Research, and the Interdisciplinary Principles and Guidelines for the Use of Animal in Research, Testing, and Education by the New York Academy of Sciences, Ad Hoc Animal Research Committee.

Rats were divided into four groups depending on the treatment provided in the drinking water (*n* = 5): Group 1, vehicle-drinking SD × LE group; Group 2, EGCG-drinking SD × LE group; Group 3, vehicle-drinking P23H group; Group 4, EGCG-drinking P23H group. EGCG (Sigma E4268, Madrid, Spain) was dissolved in drinking water at a concentration equivalent to 10 mg/kg/day, considering body weight and water consumption measured every 2 weeks. Water bottles were sealed to avoid light throughout the experiment. EGCG stock solution was prepared freshly twice per week to minimize its oxidation. Treatments started at postnatal day 21 (P21) and lasted until P180. Visual parameters were analyzed during this period to evaluate the progression of the disease.

### 2.2. Visual Assessment: Visual Acuity and Contrast Sensitivity

Rats were measured from P30 to P180 every month. VA and contrast sensitivity (CS) parameters were evaluated using an Optomotry system (OptoMotry®, CerebralMechanics, Lethbridge, AB, Canada) as already described [40]. The device is composed of a square-testing chamber formed by four screens located around a platform to place the animals. Sine-wave gratings are projected on computer monitors creating a virtual cylinder around the animal rotating at a speed of 12°/s. The experimenter judges if the rat followed the stimuli or not using the video providing real-time feedback on a computer. When the rat is not able to track the stimuli, it is assumed that it cannot see the grating and the spatial frequency threshold. This threshold is considered the maximum VA, obtained by increasing the grating spatial frequency at 100% contrast. To reduce the adaptation to the stimulus, completely grey screens appeared after 5 s of tracking movement. Each eye was measured independently depending on the direction of the rotation of the stimuli: clockwise rotation for the left eye and counterclockwise rotation to measure the right eye.

A CS curve was produced by identifying the minimum contrast that generates tracking over a range of spatial frequencies [41].

### 2.3. ERG Recordings

Rats were dark-adapted overnight at P180. Using a dim red light, animals were anesthetized using a mixture of ketamine (90 mg/kg intraperitoneal, i.p.) and xylazine (10 mg/kg i.p.) [42]. Pupils were dilated with a drop of 1% tropicamide (Alcon Labs, Barcelona, Spain) and the cornea was maintained wet using 2% methocel (OmniVision, Puchheim, Germany) and saline solution. ERGs were obtained using a gold wire loop as the recording electrode. Stimulus presentation and data acquisition were provided by the Espion® system (Diagnosys LLC, Cambridge, UK). All experiments were performed in scotopic conditions.

### 2.4. Mixed Scotopic ERG Response

Dark-adapted waves show the contributions of both photoreceptor pathways. For their characterization, recordings of 3 to 10 single-flash presentations of 10 µs duration were displayed as previously described [22]. Stimuli were presented at 10 increasing intensities varying from −3.70 to 2.86 log cd·s/m^2^. Interstimulus intervals (ISIs) were increased to avoid rod photopigment bleaching. The ISI varied from 10 s at the lowest stimulus intensity (−3.70 log cd·s/m^2^) to 120 s at the highest stimulus intensity (2.86 log cd·s/m^2^). The a-wave amplitude was measured from the baseline to the trough of the a-wave. The amplitude of the b-wave was measured from the trough of the a-wave up to the peak of the b-wave, excluding the peak of the oscillations. The results of a- and b-wave measurements were averaged from the different recordings. To determine the wave’s loss, criterion amplitudes were established at 20 µV for both a- and b-waves.

### 2.5. Isolation of the Cone Response Using a Double-Flash Protocol

The double-flash protocol was similar to that already described [43,44]. Briefly, a probe flash was presented 1 s after a first conditioning flash to avoid the rod response to the second flash due to light bleaching. Both flashes were set to 1.4 log cd·s/m^2^. The response to the probe flash with no rod contribution was considered the cone activity. The rod-driven b-wave was calculated by subtracting the cone-driven response from the mixed response. The results were calculated as the average of 3 recordings and ISI was set to 100 s to ensuring the full recovery of rod responsiveness.

### 2.6. Activity and Body Temperature Recording

Before the end of the treatment, at P180, rats were implanted with an intraperitoneal transmitter (TA-T20^®^, Data Sciences International, St. Paul, MN, USA). After surgery, animals were placed separately in cages and each cage was placed on a receiver to collect activity and temperature data every 10 min for 1 week, using specific software (Dataquest A.R.T (Data Sciences International, St. Paul, MN, USA). Collected data were analyzed using El Temps software (El Temps^®^ 1.292, Díez Noguera, University of Barcelona, Barcelona, Spain) and Circadianware^®^ (University of Murcia, Murcia, Spain). We performed a non-parametric analysis as previously described [45], including interdaily stability (IS), intradaily variability (IV), and relative amplitude (RA). The IS index describes the stability between the days, and values vary between one for rhythmic activity behaviors and zero for arrhythmic behaviors. The IV index quantifies the fragmentation of the rest–activity rhythm, producing higher values for disrupted activity patterns, such as a greater number of shorter periods, both of activity and rest, instead of one of each type of longer duration. RA is the ratio between the 10 most active hours (M10) and the 5 least active hours (L5) and is calculated using the equation: (M10 − L5)/(M10 + L5). Results nearer to 1 indicate a higher amplitude of circadian rhythm. The desynchronization index L2 correlates the 2 h of lowest temperature and the 2 h of least activity, and also the scoring ranges from 0 for an ideal synchronization to 1 for 12 h of difference among both parameters.

### 2.7. Sample Collection and Biochemical Evaluation

Six months after starting treatment, animals were euthanized by carbon dioxide asphyxiation. The liver was quickly dissected and homogenized in 0.2 M phosphate buffer composed of Na_2_HPO_4_ (Panreac, A1046, Barcelona, Spain NaH_2_PO_4_ (Panreac, A3559, Barcelona, Spain) (pH = 7.4), 0.5% Triton X-100 (Panreac, A4975, Barcelona, Spain), 5 mM β-mercaptoethanol (M6250, Sigma Aldrich, Madrid, Spain), and 0.1 mg/mL phenylmethylsulfonyl fluoride (P7626, Sigma Aldrich, Madrid, Spain) for measuring the enzyme concentrations and other biochemical parameters as previously described [22]. The tissue was homogenized for 10 min at 3000× *g* at 4 °C and the supernatants obtained were frozen at −80 °C until their analysis.

### 2.8. Total Protein Quantification

The Bradford method was used for hepatic protein quantification [46]. The sample concentration was calculated using the standard curve with bovine serum albumin as the standard protein.

### 2.9. Lipid Peroxidation

Evaluation of lipid peroxidation was used to measure the oxidative damage of the cell membranes. The main products in lipid peroxidation are malondialdehyde (MDA) and hydroxyalkenals (4-HDA); their values were used to estimate the level of lipid breakdown. Both products react with N-methyl-2-phenylindole at 45 °C, forming a colorimetric product with maximal absorbance at 586 nm proportional to the amount of MDA and 4-HDA present in samples [47]. The value of lipid peroxidation in the homogenates is expressed as nmol of MDA + 4-HDA per milligram of protein.

### 2.10. Protein Carbonyl Groups

Oxidation of proteins by free radicals produces carbonyl groups, which are markers of oxidative injury. These groups are chemically stable and can be detected when reacting with 2,4-dinitrophenylhydrazine (D199303, Sigma Aldrich, Madrid, Spain), resulting in stable 2,4-dinitrophenylhydrazone products and UV-visible spectrophotometrically at 375 nm [48]. Results are expressed as nmol of protein carbonyl groups per mg of protein.

### 2.11. Nitrosative Damage

During pathological processes and aging, nitric oxide (NO) reacts with other molecules, including superoxide anions acting as free radicals, having harmful effects. The final products of NO are nitrites (NO_2_) and nitrates (NO_3_); their sum measures NO total production. Griess reagent (03553, Sigma-Aldrich, Madrid, Spain) converts NO_2_ into a pink/purple product measured spectrophotometrically at 550 nm, which is proportional to the NO concentration present in samples [49]. The nitrite concentration is expressed as nmol of nitrate and nitrite per mg of protein.

### 2.12. GSH/GSSG Ratio

Glutathione (GSH) is a tripeptide that can be found in cells in the reduced (GSH) or oxidized (GSSG) state; it plays a key role in preventing cell damage due to free radicals. When cells are exposed to oxidative stress, GSSG accumulates and the ratio of GSH/GSSG decreases, providing a useful indicator of cellular damage. Ellman’s reagent (5,5’-dithiobis-2-nitrobenzoic acid (DNTB) (D8130, Sigma Aldrich, Madrid, Spain) reacts with sulfhydryl groups (GSH) resulting in a yellow product (5-thio-2-nitrobenzoic acid) that can be measured at 412 nm [50]. Amplifying the concentration of GSSG in samples is necessary to expose them to a thiol scavenger, 4-vinylpyridine (L13316AC, AlfaAesar Thermo Fisher Scientific, Madrid, Spain). This compound prevents GSH reacting to DTNB, making a pyridinium salt [51].

### 2.13. Total Antioxidant Capacity (TAC)

This method studies the ability of antioxidant molecules to metabolize free radicals present in cells, including small proteins and different enzymes. To form an ABTS radical (ABTS^•+^), 2,2’-azino-bis(3-ethylbenz-thiazoline-6-sulfonic acid (ABTS; A1888, Sigma-Aldrich, Madrid, Spain) can be oxidized by metmyoglobin (M1882, Sigma-Aldrich, Madrid, Spain) and hydrogen peroxide (H1009; Sigma Aldrich, Madrid, Spain). The blue-green product can be measured at 405 nm and its level is inversely proportional to the concentration of antioxidant molecules in biological samples [52]. Results are expressed in mM Trolox equivalents per mg of protein, with Trolox (238813, Sigma-Aldrich, Madrid, Spain) being a water-soluble tocopherol analogue used to standardize antioxidants.

### 2.14. Antioxidant Enzymes

The antioxidant activities of catalase (CAT), superoxide dismutase (SOD), and glutathione S-transferase (GST) were measured as previously described [22].

CAT (CAT, EC 1.11.1.6) activity of the sample was measured following the decrease in H_2_O_2_ for 30 s at 240 nm. The enzymatic activity was calculated using the molar extinction coefficient of H_2_O_2_, 43.6 cm^−1^M^−1^, and expressed in U/mg per protein. One unit of CAT activity transforms 1 μmol of H_2_O_2_ per min.

The method to measure SOD (SOD, EC 1.15.1.1) activity is based on the inhibition of the rate of reduction of cytochrome c (C2506, Sigma-Aldrich, Madrid, Spain) by O_2_^•−^, with a xanthine (X7375, Sigma-Aldrich, Madrid, Spain)/xanthine oxidase (X4500, Sigma-Aldrich, Madrid, Spain) system as a source of O_2_^•−^. Reduced cytochrome c can be observed at 550 nm. Data are presented as U/mg of protein, with one unit of SOD being the amount of enzyme that inhibits the rate of cytochrome c reduction by 50%.

GST (GST, EC 2.5.1.18) catalyzes the reaction of the sulfhydryl groups of the GSH with 1-chloro-2,4-dinitrobenzene (CDNB) (237329, Sigma-Aldrich, Madrid, Spain). The result is a conjugate GSH-CDNB, which can be detected by spectrophotometry at 340 nm. The rate of increase in absorbance is directly proportional to the GST activity in the sample. One unit of GST is defined as the amount of enzyme that catalyzes the conjugation of 1 nmol of GSH-CDNB per min.

### 2.15. Statistical Analysis

The data were plotted as the mean ± standard error of the mean (SEM). The normality test used to determine data distribution was the Shapiro–Wilk test. *p*-values less than 0.05 were considered statistically significant. The non-parametric test, the Kruskal–Wallis test, was used to evaluate differences in medians among groups; the Mann–Whitney U test was used for group comparison.

IBM SPSS Statistics 20 (IBM Corp, Armonk, NY, USA) was used for statistics, and graphs were constructed with GraphPad Prism version 7 (GraphPad Software, San Diego, CA, USA).

## 3. Results

### 3.1. Visual Parameters

**Figure 1:**
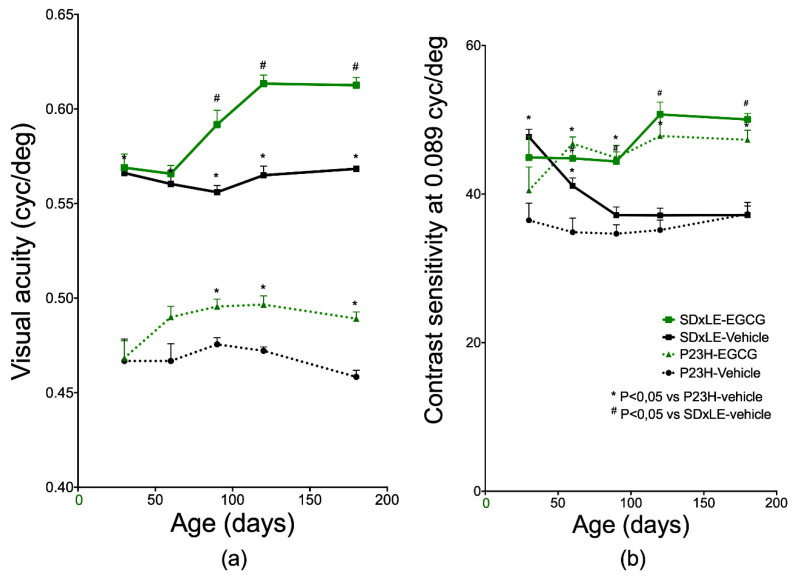
Values of (**a**) visual acuity (VA) and (**b**) contrast sensitivity (CS) at 30, 60, 90 and 180 postnatal days in P23H and Sprague–Dawley (SD) × Long Evans (LE) treatment groups, obtained by the optomotor system on average between the clockwise and anti-clockwise directions. Data represent the mean ± standard error (*n* = 5 per group). Mann–Whitney U test, * *p* < 0.05 versus P23H-vehicle animals, # *p* < 0.05 versus SD × LE animals.

At P30, VA values were 0.467 ± 0.012 cycles/degree (cyc/°) for P23H rats and 0.566 ± 0.002 for the wild-type rats untreated group. VA was progressively lost with age in P23H rats; SD × LE rats preserved the VA with time, with a VA value of 0.5684 ± 0.0007 cyc/° at P180. Statistically significant differences were observed from P90 until the end of the experiment, both in P23H and SD × LE-EGCG groups (0.489 ± 0.004 cyc/° and 0.613 ± 0.004 cyc/°, respectively, at P180; Figure 1a).

For a spatial frequency of 0.089 cyc/° (Figure 1b), peaks of 36.476 ± 2.310 and 47.704 ± 1.020 were obtained in P23H and SD × LE animals at P180 without treatment, and 47.310 ± 1.270 and 50.050 ± 0.790 in treated animals.

The CS curves showed a worsening in SD × LE rats with no treatment, with similar results to those obtained in the P23H rats (47.704 ± 1.020 and 36.476 at P30 and 37.188 ± 1.220 and 37.312 ± 1.580 at P180 in SD × LE and P23H non-treated groups, respectively). However, in both cases, EGCG treatment increased CS values at P180: 50.050 ± 0.790 in SD × LE-EGCG and 47.310 ± 1.280 in P23H-EGCG.

### 3.2. ERG Recordings

**Figure 2:**
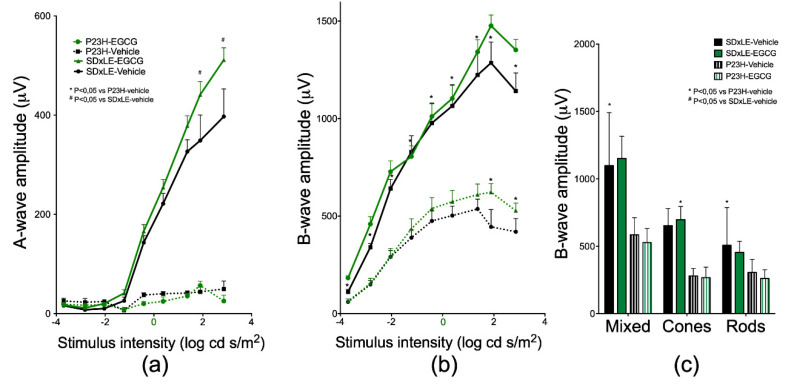
Scotopic (**a**) a- and (**b**) b-wave responses for flash intensities of 0.0002, 0.0015, 0.0092, 0.06, 0.38, 2.38, 23.19, 78, and 722 cd.s/m^2^ and electroretinogram double flash protocol (at 1.4 log cd.s/m^2^) (**c**) recorded at P180 in P23H and SD × LE treatment groups. Data are presented as mean ± standard error (*n* = 5 per group). Mann–Whitney U test, * *p* < 0.05 versus P23H-vehicle animals, # *p* < 0.05 versus SD × LE animals.

ERG recordings are presented in Figure 2.

At P180, SD × LE-vehicle rats reached a maximum value of 397.300 ± 55.800 µV at the highest stimulus intensity of 2.86 log cd.s/m^2^ in scotopic a-wave (Figure 2a). At P180, P23H-vehicle rats reached a maximum of 49.800 ± 16.100 µV. This value was 12.5% of the amplitude obtained in wild-type rats. EGCG treatment slowed the amplitude loss in SD × LE rats in which a-wave amplitude increased almost 29% (511.500 ± 24.600 µV) compared to the non-treated group with a 2.86 log cd.s/m^2^ stimulus intensity. However, P23H rats showed no changes with treatment, with values of 25.500 ± 12 µV, which were slightly lower than in the sham group. The preservation of the mean values of b-wave amplitudes (Figure 2b) was better than those of the a-waves. As already described, b-wave amplitude in P23H rats was lower than wild-type rats: 445.180 ± 88.700 µV and 1285.900 ± 106.300 µV, respectively, using a stimulus intensity of 1.89 log cd.s /m^2^. As with a-waves, EGCG treatment groups showed higher amplitudes than vehicle groups with a maximum peak of 623.100 ± 43.800 µV in SD × LE rats and 1476.300 ± 55 µV in transgenic rats.

In P23H rats, mixed b-wave amplitude statistically significantly reduced (585.320 ± 39.850 µV compared to 1098.980 ± 160.450 µV in SD × LE rats). In these animals, cone response decreased a 42.8% compared to SD × LE rats, and rod contribution was the most affected parameter, with values of 305.450 ± 30.700 µV in P23H rats and 506.800 ± 114.600 µV in wild-type animals. P23H EGCG group values were 527.800 ± 32.900 µV in mixed response, 267.100 ± 24.700 µV in cone contribution, and 260.700 ± 20.200 µV in isolated rod b-wave response, slightly lower than those obtained without treatment. In SD × LE rats treated with EGCG, the results obtained were 1152.100 ± 51.900 µV, 697.100 ± 31 µV, and 455 ± 26 µV, respectively. In all cases, there were no statistically significant differences (Figure 2c).

### 3.3. Temperature and Locomotor Rhythm

**Figure 3:**
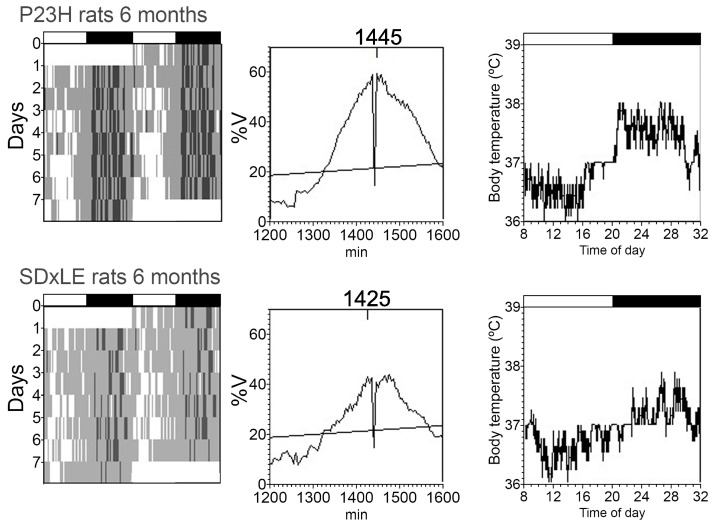
Representative actograms (left), periodograms (middle), and mean waveforms (right) obtained analyzing core-body temperature rhythms at P180 in P23H and SD × LE rats without treatment. The light/dark cycle is represented by white and dark horizontal bars, respectively.

**Figure 4:**
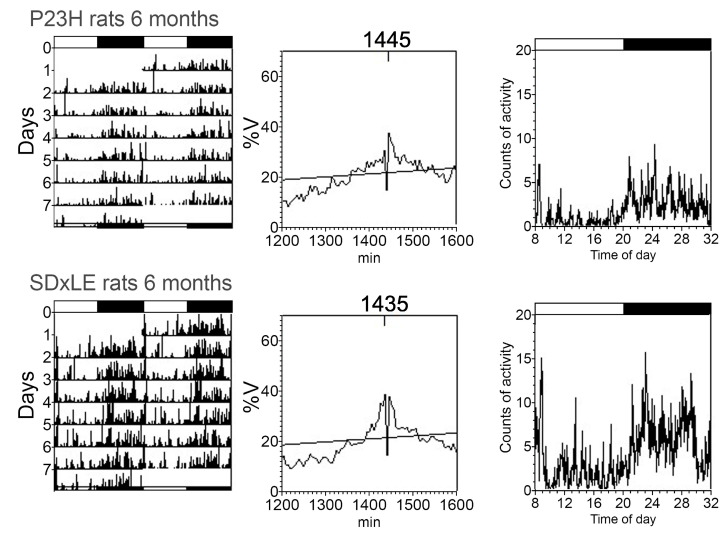
Representative actograms (left), periodograms (middle), and mean waveforms (right) obtained analyzing locomotor activity rhythms at P180 in P23H and SD × LE rats without treatment. The light/dark cycle is represented by white and dark horizontal bars, respectively.

**Figure 5:**
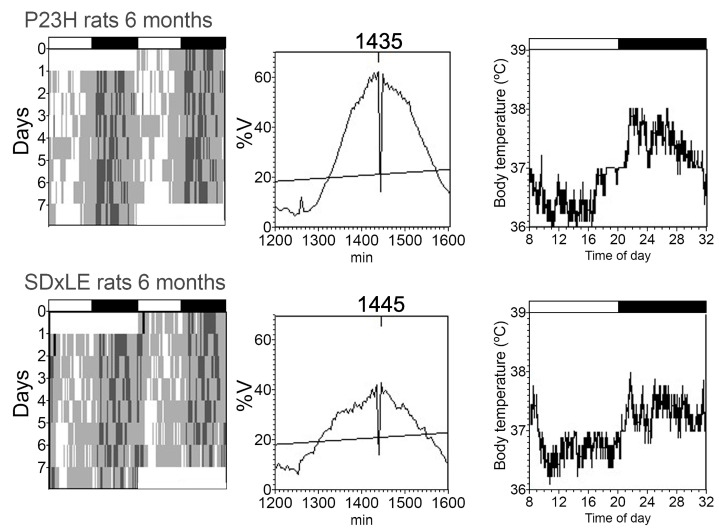
Representative actograms (left), periodograms (middle), and mean waveforms (right) obtained analyzing core-body temperature rhythms at P180 in P23H and SD × LE rats with epigallocatequin gallate (EGCG) treatment. The light/dark cycle is represented by white and dark horizontal bars, respectively.

**Figure 6:**
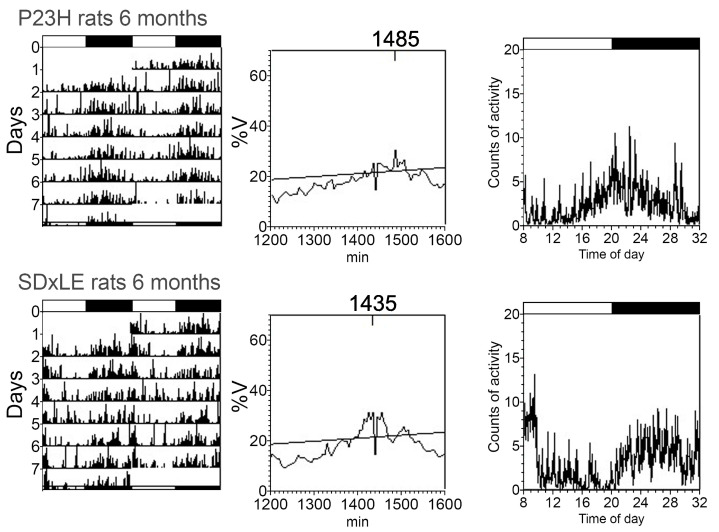
Representative actograms (left), periodograms (middle), and mean waveforms (right) obtained analyzing locomotor activity rhythms at P180 in P23H and SD × LE rats with epigallocatequin gallate (EGCG) treatment. The light/dark cycle is represented by white and dark horizontal bars, respectively.

An example of actograms, periodograms, and mean waveforms of core-body temperature and locomotor activity rhythms is shown in Figure 3 and Figure 4 without treatment and with EGCG treatment in Figure 5 and Figure 6 at P180 for a P23H and SD × LE rat exposed to a 12:12 light/dark cycle. Data were registered for seven days and were obtained from the same animal.

Table 1 shows the comparative data recorded from transgenic P23H and SD × LE rats. The most significant difference between untreated groups was a difference of almost nine hours in the two lowest-temperature hours (L2) comparing SD × LE (5:05 ± 2:30 h) to P23H rats (13:55 ± 1:24 h). We found significant differences in the same parameter related to activity but with a smaller difference (6:31 ± 0:24 h in SD × LE rats vs. 6:45 ± 0:20 h in P23H rats). The P23H rats exhibited worse temperature–activity synchronization than SD × LE rats, with desynchronization index L2 values of 0.60 ± 0.11 and 0.19 ± 0.14, respectively, indicating a seven-hour window between the start time of two hours of minimum temperature and the start time of two hours of minimum activity in transgenic P23H animals.

Control rats showed higher values of locomotor activity (4.47 ± 0.42) compared to P23H rats (2.69 ± 0.28). The analysis of non-parametric variable IV evidenced a slightly increased rhythm fragmentation in P23H rats. In SD × LE rats, the main changes with EGCG administration were decreased desynchronization index L2 (0.09 ± 0.04, *p* < 0.05) and a lower value of CFI temperature index, which is a variable based on the previous three parameters, IS, IV, and RA, which represent worse circadian rhythmicity in treated animals.

Treatment with EGCG in P23H rats significantly decreased the acrophase value in locomotor activity (982.23 ± 7.71 compared to 1056.88 ± 21.28 in untreated P23H rats) and IS non-parametric variable. EGCG-treated P23H showed a worse circadian rhythm than the sham group.

### 3.4. Oxidative Stress Parameters

**Figure 7:**
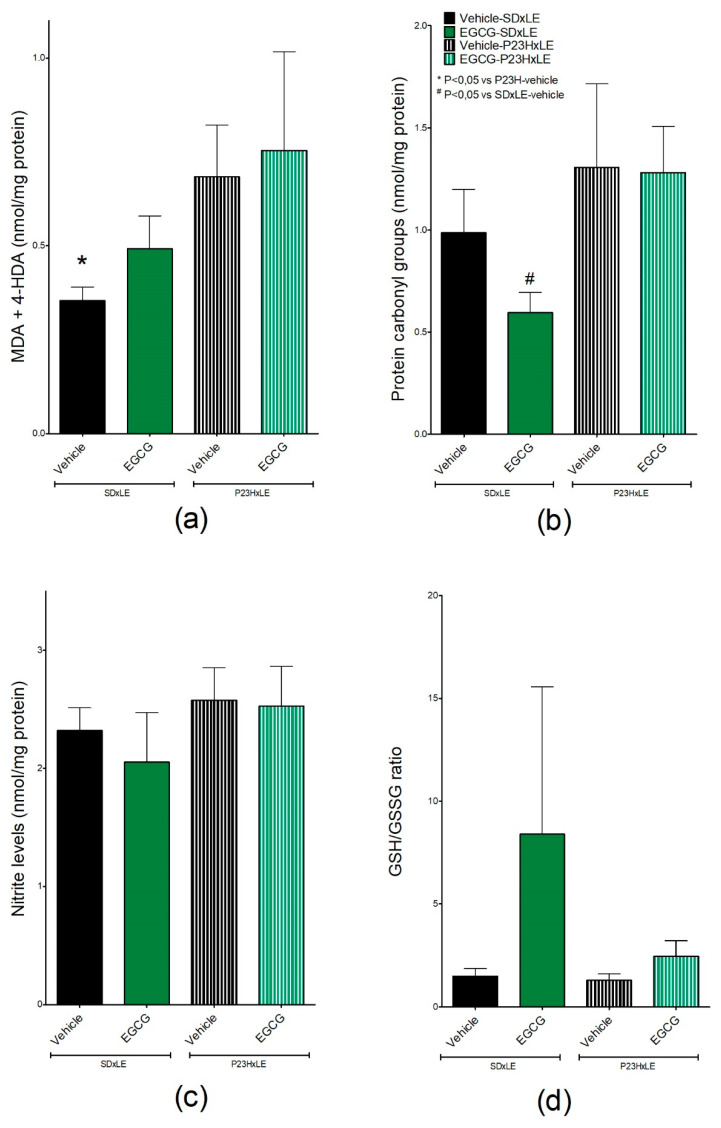
Levels of (**a**) malondialdehyde and 4-hidroxyalkenals (MDA and 4-had, respectively), (**b**) oxidized proteins (protein carbonyl groups), (**c**) nitrosative damage (nitrite levels), and (**d**) the ratio of reduced and oxidized glutathione (GSH/GSSG) in the liver of P23H and SD × LE treatment groups. Epigallocatequin gallate (EGCG). Data represent the mean ± standard error (*n* = 5 per group). Mann–Whitney U test, * *p* < 0.05 versus P23H-vehicle animals, # *p* < 0.05 versus SD × LE animals.

MDA and 4-HDA values, the main products of lipid peroxidation and oxidative stress markers that were measured in the livers of P23H-vehicle rats, were significantly higher (0.684 ± 0.1 nmol/mg protein) than in SD × LE-vehicle (0.354 ± 0.04 nmol/mg protein), expressing increased levels of reactive oxygen species (ROS) in these animals. Both P23H-EGCG and SD × LE-EGCG exhibited higher levels (0.754 ± 0.2 and 0.492 ± 0.09 nmol/mg protein, respectively), but these results did not reach statistically significant differences (Figure 7a).

Levels of oxidized proteins were measured by protein carbonyl groups (Figure 7b), another biomarker of oxidative stress. SD × LE-EGCG showed lower levels of protein damage than SD × LE-vehicle (0.595 ± 0.1 nmol/mg protein and 0.987 ± 0.2 nmol/mg protein, respectively) opposite to our findings in P23H-EGCG, in which values were similar than those obtained in P23H-vehicle (1.282 ± 0.2 nmol/m protein vs. 1.306 ± 0.4 nmol/mg protein, respectively). Although carbonyl levels in P23H rats were higher than in SD × LE rats, differences did not reach statistical significance.

Hepatic nitrite levels were measured as an index of NO production and therefore nitrosative damage (Figure 7c). There was a trend of lower nitrite levels in groups treated with EGCG compared to sham groups, with no significative differences (0.252 ± 0.3 nmol/mg protein in P23H-EGCG versus 0.258 ± 0.3 nmol/mg protein in P23H-vehicle and 2.052 ± 0.4 nmol/mg protein in SD × LE-EGCG compared to 2.319 ± 0.2 nmol/mg protein). Comparing non-treated groups, we found slightly higher levels in transgenic rats than in wild type, with no significant differences.

The ratio GSH/GSSG is an indicator of cellular health. Values were similar between P23H and SD × LE rats without treatment (1.29 ± 0.3 and 1.49 ± 0.4, respectively). EGCG increased this ratio in both groups (2.46 ± 0.7 in P23H rats and 8.40 ± 7.2 in SD × LE) with no statistically significant differences (Figure 7d).

### 3.5. Antioxidant Parameters

**Figure 8:**
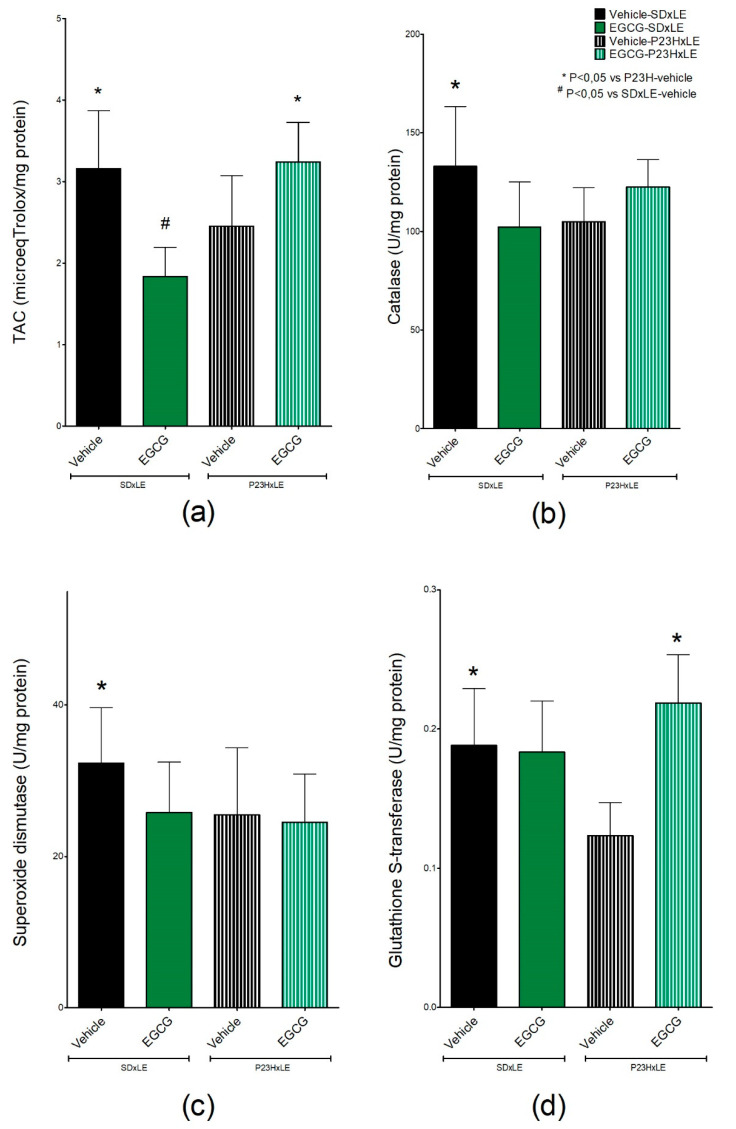
Levels of (**a**) total antioxidant capacity (TAC), (**b**) catalase (CAT), (**c**) superoxide dismutase (SOD), and (**d**) glutathione S-transferase (GST) activity in P23H rats’ livers and SD × LE treatment groups. Epigallocatequin gallate (EGCG). Data are represented as the mean ± standard error (*n* = 5 per group). Mann–Whitney U test, * *p* < 0.05 versus P23H-vehicle animals, # *p* < 0.05 versus SD × LE animals.

Measurement of total antioxidant capacity (Figure 8a) revealed statistically significantly higher levels of antioxidant defenses in wild-type rats (3.16 ± 0.7 µeq Trolox/mg protein) than in P23H rats (2.45 ± 0.6 µeq Trolox/mg protein). In EGCG-treated groups, SD × LE exhibited lower values compared to the untreated group (1.84 ± 0.4 µeq Trolox/mg protein), whereas P23H rats had higher antioxidant levels (3.24 ± 0.5 µeq Trolox/mg protein).

The activities of three of the main antioxidant enzymes, CAT, SOD, and GST, were lower in P23H rats than in SD × LE rats. GST (Figure 8d) was the only enzyme that achieved significant differences in treated P23H rats (0.124 ± 0.02 U/mg protein in the sham group and 0.219 ± 0.03 U/mg protein in P23H-EGCG). There were no differences between CAT and SOD activity with or without treatment (Figure 8b,c, respectively).

## 4. Discussion

Our study focused on the effect of the main catechin of green tea, EGCG, administered to an animal model of RP, the P23H rat. Previous studies demonstrated the ability of EGCG to reach retinal tissue and act as an antioxidant, diminishing levels of oxidative stress and apoptosis and improving visual function [29,38,53,54,55]. The P23H rat was created by the incorporation of a mutated rhodopsin transgene from C57BL/6J mouse in an albino wild-type rat (Sprague–Dawley (SD)) resulting in a homozygous transgenic animal. These rats were crossed with pigmented LE rats to obtain heterozygous pigmented rats to resemble patients carrying this mutation [5,39]. Due to the SD background, we chose the wild-type animal SD × LE to include the effects of the albino background in the model. The administration of EGCG to SD × LE rats was intended to verify the long-term effect of this compound on healthy individuals. The loss of visual function evaluated with VA and CS by optomotor in P23H over six months is supported by numerous studies conducted in this animal model [6,39,41,56]. Rhodopsin mutation is responsible for the apoptosis of rod and cone photoreceptors, triggering general disorganization and remodeling of the whole retina with the subsequent visual field and VA losses, ending in blindness in the final stage [57,58].

EGCG is a polyphenol with a low molecular weight and hydrophilic properties, which allow it to reach ocular tissues, including the retina, and exert biological effects [26]. Its administration to P23H and SD × LE rats delayed the neurodegenerative and aging processes in these animals, with higher values of both visual parameters, VA and CS, compared to non-treated groups, probably due to a decrease in levels of oxidative stress, which were demonstrated to play an important role in neurodegeneration [59,60,61,62,63]. EGCG may reduce levels of free radicals present in the retina [64,65,66,67], slowing the advance of the RP and age-related changes.

Electrophysiological results obtained by full-field ERG in P23H and control rats are similar to those previously published [5,41]; in scotopic a-wave amplitude, the administration of EGCG is not able to slow the amplitude loss. SD × LE treated rats showed an increase in the amplitude values compared to the sham group, probably as a result of its antioxidant properties. B-wave amplitude results are supported by other studies [36], which reported that the injection of EGCG in an animal model of light-induced retinal degeneration protected the retina, attenuating the loss of visual function and preserving its structure. An explanation could be the presence of an EGCG receptor on the cell surface, the non-integrin laminin receptor 67LR, in some of the retinal cells such as glial cells, which participate in the inflammation process [68]. The results obtained with the double-flash protocol at 1.4 log cd.s/m^2^ showed similar responses of both rods and cones, which indicated that the cones of P23H rats were affected by the disease and EGCG was not able to attenuate the effect of the mutation. However, in the analysis of the scotopic b-wave, we identified higher amplitudes in EGCG-treated groups at higher stimulus intensities.

One of the non-visual functions of the retina is the synchronization of circadian rhythms. In the absence of light, the circadian system loses the regulation with the environment and acquires its own rhythm, which might not coincide with the 24-h day/night cycle. It has been reported in numerous sleep disorders and free-running cycles in blind people, which are more severe than people who maintain light perception [69]. In our study, there was a nine-hour displacement in the two lowest-temperature hours (L2) between P23H and control rats. Previous studies of the circadian rhythms in this animal model of RP showed that the retinal degeneration produced in rats P23H-1 is responsible for disrupted circadian rhythms after 24 months of disease progression, causing impairments in temperature and activity locomotor rhythms [23]. The lower locomotor activity values in the EGCG-treated group were in accordance with other studies that demonstrated the sedative, anti-dopaminergic, and hypnotic effects of this catechin-binding GABA receptor in the brain [70,71]. The effect of EGCG on circadian rhythms is limited to nocturnal animals such as rodents due to its lower influence in some neurotransmitters, e.g., dopamine. Although the antioxidant properties could exert a preventive action on retinal degeneration, it might not be enough to trigger changes in circadian rhythms as they are well-maintained until the last stages, even in cases of blindness.

Numerous studies suggested that oxidative stress is involved in pathogenesis and progression of neurodegenerative diseases such as RP. Following this idea, antioxidant therapy may be useful as a complementary treatment to slow the degeneration of retinal cells. It was previously demonstrated [22] that P23H rats are characterized by higher levels of oxidative stress than wild-type animals and that these values increase with age.

Many studies reported the antioxidant properties of EGCG and its benefits in diseases characterized by oxidative stress and diminishing levels of lipid peroxidation [72,73,74,75,76]. However, this molecule is also known for its pro-oxidant effects, being responsible, in some cases, for hepatic damage due to increased levels of free radicals and inflammation in both animal models and human patients [77,78,79,80,81]. In our study, EGCG-treated groups showed higher levels of MDA and 4-HDA compared to the sham group, although the concentration administered was so much lower than in other experiments in which authors reported negative effects. Levels of nitrites and carbonyl groups, as indicators of nitrosative damage and a protein marker of oxidative stress, respectively, were similar when comparing EGCG-treated and non-treated P23H groups. EGCG reduced levels of both biomarkers in control animals, which were reported to increase with age, probably by modulation of nitric oxide synthase enzymes (NOS) and its free radical scavenger activity [82,83,84,85,86]. EGCG was shown to protect the GSH/glutathione peroxidase system [87], increasing or maintaining the ratio GSH/GSSG, and thereby improving the oxidative status of treated rats [26].

Based on what is known about EGCG, this catechin may modulate antioxidant systems by activating the nuclear factor erythroid-2-related factor 2 (Nrf2) antioxidant response element pathway [88,89,90]. In the EGCG-treated P23H group, levels of TAC were higher, but not in the SD × LE group, where animals showed a decrease compared to those without treatment. All antioxidant markers analyzed were lower in P23H rats than in wild-type rats; however, it is still unclear if the overproduction of free radicals is the result of this decrease in antioxidant systems and if it is a cause or a consequence of the disease [91,92,93].

The only enzyme with increased activity after EGCG administration was GST. Nrf2 is a cytoplasmic protein involved in the expression of detoxifying and antioxidant enzymes, including GST, so its modulation is important in neurodegenerative disease to slow its progression. Han et al. [94] reported the ability of EGCG to inhibit hepatotoxicity induced by arsenic, by activating the Nrf2 signaling pathway. Although studies reported the influence of EGCG in CAT and SOD activities [95,96,97,98], we did not find any differences between treated and untreated groups.

As the antioxidant and pro-oxidant activity of EGCG is not well established yet due to variation related to genetic background [99], doses, and pathological conditions [100], its use as a long-term treatment or supplement must be treated with caution.

## 5. Conclusions

In conclusion, we found that EGCG improves visual function and hepatic oxidative stress in P23H rats, probably due to its antioxidant and anti-inflammatory properties. Visual improvements were found in control animals too, but not those related to oxidative damage, in which it was possible to identify a loss of antioxidant defenses. We could not establish a clear conclusion in relation to the EGCG effect in circadian rhythms. Further research is necessary to elucidate the different molecular mechanisms of action of this catechin and to determine its long-term effects in both disease and health conditions.

## Figures and Tables

**Table 1:** Circadian parameters.

	SD × LE				P23H × LE			
	Temperature		Locomotor Activity		Temperature		Locomotor Activity	
	Vehicle (*n* = 5)	EGCG (*n* = 5)	Vehicle (*n* = 5)	EGCG (*n* = 5)	Vehicle (*n* = 5)	EGCG (*n* = 5)	Vehicle (*n* = 5)	EGCG (*n* = 5)
Rhythm parameters								
Mesor (°C)	36.96 ± 0.04	36.99 ± 0.04	4.47 ± 0.42 *	3.14 ± 0.21 #	37.16 ± 0.13	36.96 ± 0.03	2.69 ± 0.28 #	2.67 ± 0.21
Amplitude (°C)	0.37 ± 0.01 *	0.46 ± 0.10	2.67 ± 0.35	2.24 ± 0.24	0.51 ± 0.06 #	0.56 ± 0.05	1.90 ± 0.17	1.60 ± 0.10
Acrophase (min)	1118.26 ± 23.66	1088.46 ± 11.86	1145.71 ± 35.07	1175.15 ± 30.04	1022.90 ± 30.69	1005.43 ± 14.16	1056.88 ± 21.28	982.23 ± 7.71 *
Acrophase (hh:mm)	2:43 ± 0:23	2:13 ± 0:11	3:10 ± 0:35	3:40 ± 0:30	1:07 ± 0:30	0:50 ± 0:14	1:41 ± 0:21	0:27 ± 0:07 *
Variance (%)	26.08 ± 1.076 *	30.49 ± 7.936	11.77 ± 1.59	14.04 ± 2.18	38.03 ± 4.806 #	42.05 ± 4.473	13.24 ± 1.39	8.40 ± 0.49 *
Period (min)	1445.00 ± 20.00	1440.00 ± 5.00	1441.67 ± 3.33	1445.00 ± 4.08	1442.50 ± 2.50	1445.00 ± 4.08	1442.50 ± 2.50	1460.00 ± 8.66 *
Non-parametric variables								
IS	0.63 ± 0.03	0.61 ± 0.03	0.33 ± 0.02	0.35 ± 0.03	0.69 ± 0.04	0.68 ± 0.02	0.32 ± 0.03	0.23 ± 0.01 *
IV	0.25 ± 0.03	0.21 ± 0.02	0.96 ± 0.01	0.91 ± 0.09	0.18 ± 0.03	0.18 ± 0.02	1.08 ± 0.07	1.08 ± 0.04
RA	0.01 ± 0.00	0.01 ± 0.00	0.50 ± 0.02	0.64 ± 0.03 #	0.01 ± 0.00	0.01 ± 0.00	0.60 ± 0.04	0.51 ± 0.04
L2 (hh:mm)	5:05 ± 2:30 *	6:35 ± 0:21	6:31 ± 0:24 *	6:27 ± 0:13	13:55 ± 1:24 #	12:42 ± 0:56	6:45 ± 0:20 #	6:30: ± 0:33
VL2 (°C)	37.53 ± 0.08	37.57 ± 0.07	4.67 ± 0.89 *	3.06 ± 0.25	37.43 ± 0.07	37.29 ± 0.04 *	2.82 ± 0.13 #	2.27 ± 0.16
Media (°C)	37.43 ± 0.04	37.45 ± 0.04	4.87 ± 0.43 *	3.51 ± 0.21 #	37.48 ± 0.03	37.45 ± 0.02	3.07 ± 0.29 #	3.06 ± 0.21
CFI	0.30 ± 0.00	0.28 ± 0.01 #	0.60 ± 0.01	0.63 ± 0.02	0.29 ± 0.01	0.29 ± 0.00	0.66 ± 0.03	0.61 ± 0.02
DesynchroInd L2	-	-	0.19 ± 0.14 *	0.02 ± 0.01	--	-	0.60 ± 0.11 #	0.52 ± 0.08

Circadian parameters of core body temperature and locomotor activity recorded after 6 months of treatment from P23H and SD × LE rats. Mesor, 24-h time series mean; Amplitude, one-half the peak-to-trough variation of the 24-h rhythm; Acrophase, peak time relative to maximum activity/temperature; IS, interdaily stability; IV, intradaily variability; RA, relative amplitude; L2, the least temperature 2-h; CFI, circadian function index; DesynchroInd L2, desynchronization index L2. * *p* < 0.05 versus P23H-vehicle; # *p* < 0.05 versus SD × LE-vehicle.
